# *Crocodylus porosus* Sera a Potential Source to Identify Novel Epigenetic Targets: In Silico Analysis

**DOI:** 10.3390/vetsci9050210

**Published:** 2022-04-25

**Authors:** Ruqaiyyah Siddiqui, Jibran Sualeh Muhammad, Sutherland K. Maciver, Naveed Ahmed Khan

**Affiliations:** 1 College of Arts and Sciences, American University of Sharjah, Sharjah 26666, United Arab Emirates; rsiddiqui@aus.edu; 2 Department of Basic Medical Sciences, College of Medicine, University of Sharjah, Sharjah 27272, United Arab Emirates; dr.jibran@live.com; 3 Department of Clinical Sciences, College of Medicine, University of Sharjah, Sharjah 27272, United Arab Emirates; 4 Centre for Discovery Brain Science, Edinburgh Medical School, Biomedical Sciences, University of Edinburgh, Edinburgh EH8 9XD, Scotland, UK; smaciver@staffmail.ed.ac.uk

**Keywords:** epigenetics, DNA methylation, *Crocodylus porosus*, novel therapeutics

## Abstract

We have previously found that sera from *Crocodylus porosus* contain anticancer agents and the treatment of MCF7 cells with this serum resulted in the differential expression of 51 genes. The purpose of this study was to use in silico analysis to identify genes that might be epigenetically modulated in cells treated with crocodile serum and to understand the role of potential genes as novel candidates with epigenetic therapeutic potential. The findings report five proto-oncogenes (*TUBA1B*, *SLC2A1*, *PGK1*, *CCND1*, and *NCAPD2*) and two tumor suppressor genes (*RPLP2*, *RPL37*) as novel therapeutic targets. Furthermore, we present a comprehensive overview of relevant studies on epigenetic regulation of these genes along with an insight into their clinical implications. Therefore, elucidating the molecules present in the serum and gut bacteria of reptiles such as crocodiles may offer insights into the role of these genes on longevity, health, disease, and life expectancy.

## 1. Introduction

Crocodiles have shown the ability to adapt, evolve, and survive successfully over millions of years, suggesting that we ought to learn from these species. Having visited several crocodile farms and sanctuaries, we find it intriguing that animals such as crocodiles tolerate being routinely exposed to radiation, heavy metals, poor diet, pollution, etc., and have survived the catastrophic Cretaceous-Tertiary extinction event, which was the sudden mass extinction of three-quarters of the plant and animal species on Earth that happened approximately 66 million years ago. Yet crocodiles have an extended lifespan of up to 100 years and thrive in these environments which would be unfavourable to humans [1]. Furthermore, crocodiles have also been reported to possess bioactive peptides that revealed anti-oxidative, anti-inflammatory, anti-microbial, and anti-cancer attributes [1,2]. In this regard, we have recently postulated and shown that the sera and gut bacteria of animals such as crocodiles (*Crocodylus palustris* and *Crocodylus porosus)* and water monitor lizard (*Varanus salvator*), tortoise (*Cuora amboinensis kamaroma*) as well as other species that reside in polluted environments depict anti-tumour and antibacterial activities and may contribute to the longevity and hardiness of these species [3,4].

Recently, several studies have shown that epigenetics, or the study of heritable phenotypic changes, not dependent on DNA sequences, might play a pivotal role in a variety of diseases, for example, neurological disorders, cardiovascular diseases, metabolic diseases, and cancer [5]. Furthermore, epigenetic drugs are already being utilized for some cancers and neurological disorders. Several genetic alterations, such as specific gene mutations and chromosomal aberrations that are associated with breast carcinogenesis have been well studied. In addition to these highly characterized mutations, cancer initiation and progression are driven by the combined action of multiple epigenetic alterations [6]. The three well-known epigenetic alterations are DNA methylation, histone modification, and non-coding microRNA-induced modulation of gene expression. Of these, DNA methylation is the least complex and the most extensively studied. Hypermethylation of nucleotide base cytosine found before guanine, CpG Island (CGI), in the promoter regions of a tumor suppressor gene, directly blocks the binding of transcription factors essential for gene expression, thus rendering loss of expression or gene silencing [6]. However, hypomethylation of the promoter region of an oncogene could lead to its overexpression and abnormal cell survival [7]. Such alterations might be participating in the initiation and progression of breast carcinogenesis and can be useful as biomarkers for early detection of potential therapeutic targets.

The purpose of this study was to use in silico analysis to identify genes that might be epigenetically modulated in cells treated with crocodile serum and to understand the role of potential genes as novel candidates with epigenetic therapeutic potential.

## 2. Methods

### 2.1. In-Silico Analysis

All the differentially expressed genes (DEGs) in MCF-7 cells treated with crocodile serum were selected from our previous study [8]. The MCF-7 cell lines (RRID: CVCL_0031) originated from a female patient with breast adenocarcinoma and are ER-positive, PGR-positive, HER2-negative (source: Cellosaurus; https://web.expasy.org/cellosaurus/CVCL_0031; accessed on 15 February 2022). Using the criteria described previously and explained in the results [9], the initial list of 51 genes was filtered for the presence of CGIs in their promoter region. Briefly, the key genes were filtered on the basis of the presence of CGI at least 200 bp upstream of the transcription start site with GC content of >50%, length >200 bp, and ratio >0.6 of observed/expected number of CG dinucleotides and on the basis of the number of Gs and Cs in the DNA segment. A total of 24 genes were selected, which are suspected to be epigenetically regulated. Gene set enrichment analysis was performed using several ontological resources to identify the underlying pathways, and functional protein-protein interaction was depicted. Later, “Genotype-Tissue Expression (GTEx) online portal (https://www.gtexportal.org/home/; accessed on 15 February 2022)” was used to check the expression of our select genes in normal breast tissue. Lastly, for analysis of candidate gene expression and promoter methylation in cancer tissue, the “TCGA online portal (https://portal.gdc.cancer.gov/; accessed on 15 February 2022)” was accessed. Gene expression values were reported as transcripts per million (TPM) and methylation levels were presented as beta value. The beta values (*β*) are the estimate of methylation level using the ratio of intensities between methylated and unmethylated alleles. *β* are between 0 and 1 with 0 being unmethylated and 1 fully methylated.

### 2.2. Literature Review

We present a comprehensive literature review of the genes affected by DNA methylation that might lead to breast carcinogenesis, using the methods as described previously [10]. Briefly, the Medical Subject Headings (MeSH) terms used included “breast cancer” with “epigenetic,” “DNA methylation,” in combination with the names of candidate genes. We searched SCOPUS, PubMed, Ovid, Web of Science, and Google scholar databases. All the relevant studies, that were published in the last ten years, were identified and included based on their relevance.

## 3. Results

### 3.1. Crocodylus Porosus Serum-Induced Differentially Expressed Targets Genes in MCF-7 Cells

**Figure 1:**
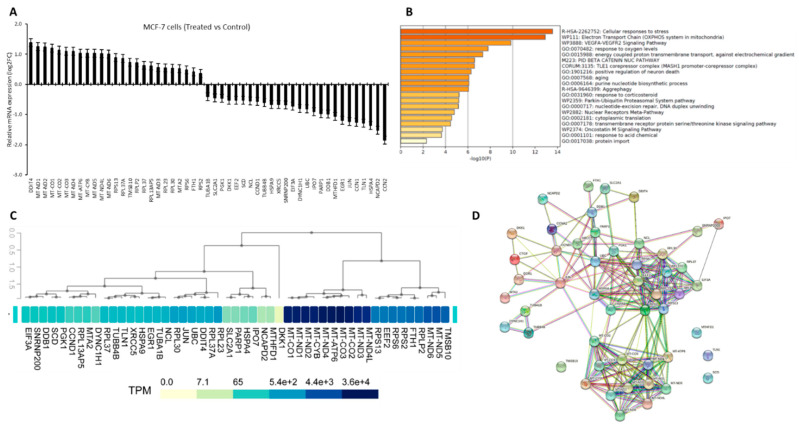
*Crocodylus porosus* serum-induced differentially expressed targets genes in breast cancer cells. (**A**). Relative mRNA expression of 51 differentially expressed genes (DEGs) in breast cancer cells treated with crocodile serum versus the control untreated cells. All plotted genes were statistically significant in comparison to the control. (**B**). Pathway enrichment analysis using DEGs as input genes. (**C**). Heatmap showing expression of DEGs in normal human breast tissues from GTEx. (**D**). Diagram showing protein-protein interactions amongst the DEGs encoded proteins.

We have previously reported a differential expression of 51 genes in MCF7 cells treated with *Crocodylus porosus* serum [8]. Here we present the relative mRNA expression of those genes as compared with untreated control cells (Figure 1A, Table S1). In these treated MCF7 cells 26 genes were downregulated, of which the *CCN2* gene was most significantly inhibited (*p* < 0.05). And 25 genes were upregulated, of which *DDIT4* was most significantly overexpressed (*p* < 0.05). Pathway enrichment analysis of our list of DEGs showed significant enrichment of GO pathways related to response to stress and electron transport genes (Figure 1B, *p* < 0.01). This indicated a strong induction of stress-related genes and mitochondrial genes-related oxidative phosphorylation. Next, we analyzed the GTEx database to investigate the expression of these in normal breast tissue. We found that most of the mitochondrial genes (such as *MT-ND1-4*, and *MT-CO1-3*) that were upregulated in serum treated MCF7 cells were already very highly expressed in the normal breast tissue. Besides, most of the genes that were significantly downregulated in serum treated cells, their expression was low in normal breast tissue. However, few of the iron regulatory genes (*FTH1* and *SLC2A1*) and ribosomal regulatory genes (*RPLP2* and *RPL37*) were expressed in contrast to normal tissues versus serum-treated MCF7 cells (Figure 1C). Then we sought to investigate the relationship between the proteins coded by these candidate genes, and STRING was used for the P-P interaction modeling. Computational prediction of direct and indirect functional interaction showed that all the candidate proteins, except for four of them, have a significant level of interaction with each other. But interestingly, the mitochondrial proteins and ribosomal proteins appeared as interacting more significantly with similar proteins (Figure 1D). Suggesting a strong effect of *Crocodylus porosus* serum on targeting these molecules.

### 3.2. Gene Filtration for Potential Epigenetic Regulation in Breast Cancer

**Figure 2:**
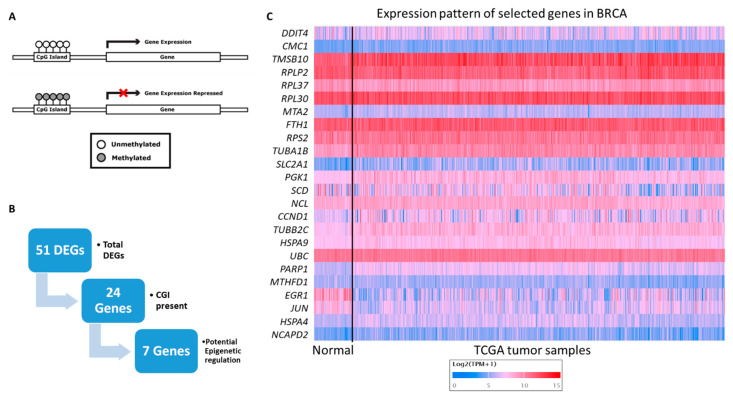
Gene filtration for identification of potential epigenetic regulation in breast cancer. (**A**). Line diagram showing DNA methylation induced epigenetic modulation of genes. (**B**). Flow chart showing the process of potential epigenetic target gene filtration. (**C**). Heatmap showing the expression pattern of 24 target genes with CpG islands (CGI) in their promoter regions in normal versus TCGA breast adenocarcinoma (BRCA) tissue samples.

DNA methylation levels are directly regulating the protein expression (Figure 2A). Therefore, firstly we aimed to investigate whether DNA methylation-based epigenetic modulation is involved in the regulation of these 51 candidate genes. So, we searched the NCBI database for the presence of CGI in their promoter regions DNA sequence upstream of the transcription start site (GC content of >50%, length >200 bp, and ratio >0.6 of observed/expected number of CG dinucleotides). Out of the 51 genes, 24 genes are having CGI in their promoter region (Figure 2B,C).

### 3.3. Novel Genes Which Might Be the Target of Crocodile Serum

**Figure 3:**
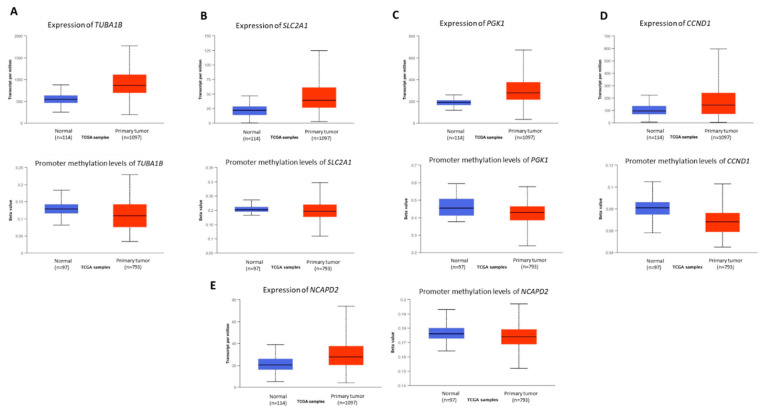
Relative mRNA expression and promoter methylation levels of the genes upregulated in cancer cells but downregulated by crocodile serum. (**A**). *TUBA1B*, (**B**). *SLC2A1*, (**C**). *PGK1*, (**D**). *CCND1*, (**E**). *NCAPD2*. (Note: for all the box-plots, *p* < 0.001).

Further, we analyzed the TCGA breast cancer datasets to check the expression of these genes in tumour versus normal tissues. Based on the gene expression and promoter methylation levels (upregulation and hypomethylation or downregulation and hypermethylation), seven (*RPLP2*, *RPL37*, *TUBA1B*, *SLC2A1*, *PGK1*, *NCAPD2*, and *CCND1*) genes were filtered as highly likely to be epigenetically regulated and a potential epigenetic target of active anticancer molecules presents in *Crocodylus porosus* serum (Figure 2B). The names and functions of the shortlisted genes which might be epigenetically regulated via crocodile serum in breast cancer cells are presented in Table 1 and Supplementary Table S1. *TUBA1B*, *SLC2A1*, *PGK1*, *CCND1*, and *NCAPD2* have been overexpressed in breast cancer tissues and their promoter region was hypomethylated (Figure 3A–E; The graph on top shows gene expression and the graph at the bottom shows methylation beta value). Suggesting an epigenetic mediated tumor-promoting function of these genes.

**Table 1:** Details of the genes highly likely to be epigenetically regulated in crocodile serum treated breast cancer cells.

Gene Symbol	Genamee n	Function (genecards.org)	Reference(s) Related to Breast Cancer
*TUBA1B*	Tubulin Alpha 1b	Involved in cell cycle spindle assembly and chromosome separation	[11,12,13,14,15]
*SLC2A1*	Solute Carrier Family 2 Member 1	Glucose transporter responsible for constitutive glucose uptake	[16,17,18,19,20,21,22]
*PGK1*	Phosphoglycerate Kinase 1	Participates in energy production via glycolysis and tumor cell angiogenesis	[23,24,25,26,27]
*NCAPD2*	Non-SMC Condensin I Complex Subunit D2	Required for conversion of interphase chromatin into mitotic-like condense chromosomes	[28,29]
*CCND1*	Cyclin D1	Progression of the cell cycle and to induce the Warburg effect in cancer cells	[30,31]
*RPLP2*	Ribosomal Protein Lateral Stalk Subunit P2	An important role in the elongation step of protein synthesis	[32]
*RPL37*	Ribosomal Protein L37	Involved in rRNA processing in the nucleus and cytosol	[33]

**Figure 4:**
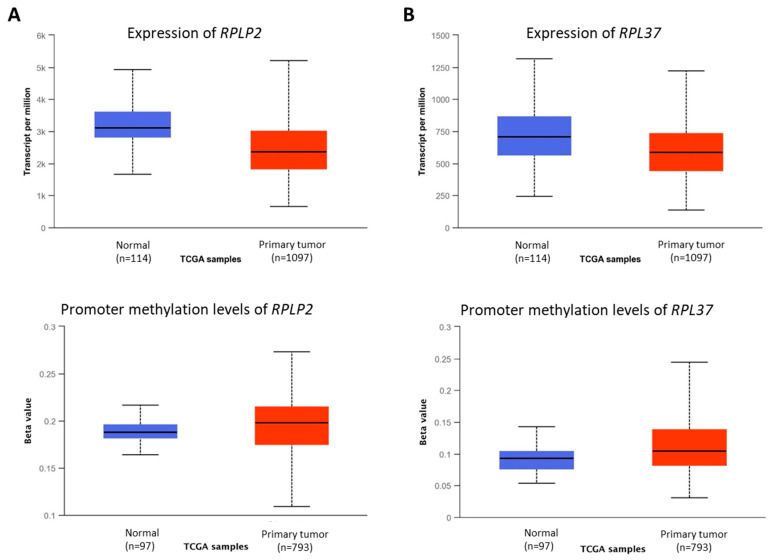
Relative mRNA expression and promoter methylation levels of the genes downregulated in cancer cells but upregulated by crocodile serum. (**A**). *RPLP2*, (**B**). *RPL37*. (Note: for all the box-plots, *p* < 0.001).

In breast cancer tissues, *RPLP2* and *RPL37* were found to be downregulated and their promoter region was hypermethylated (Figure 4A,B). Both genes were significantly up-regulated in MFC7 cells treated with crocodile serum (Figure 1A), and crocodile serum treated MCF7 cells showed reduced proliferation and decreased survival [8]. Hence, this suggests that these genes might have an epigenetic mediated tumor suppressor function. Based on these findings, we believe that these genes and their encoded proteins could be novel candidates for targeted therapy for breast cancer patients using novel molecules from crocodile serum.

## 4. Discussion

Epigenetic treatment holds great potential in the treatment of several diseases such as metabolic disorders, cancer, and cardiovascular and neurological diseases [4]. Furthermore, the ability of the epigenome to be flexible, has enabled scientists to investigate its reversal through designing epigenetic drugs as an approach to ameliorate disease phenotypes. For example, recent research has revealed that histone modifications are linked to CpG nucleotide methylation in DNA, consequently linking several epigenetic modifications and regulations [34]. With increasing research, therapeutic drug targets continue to be identified and developed with more specificity, and greater regulation of epigenetic changes may be possible. Many anticancer drugs induce programmed cell death but cellular resistance towards many of those chemotherapeutic agents has been associated with mutations/deletions of target genes. Hence it is essential to search for novel target genes to avoid the toxicity of resistant chemotherapy and the unnecessary burden of adverse effects on patients. In the search for novel targets, we analyzed the expression profile in breast cancer cells treated with crocodile serum. We have utilized an in silico approach integrating several human genome datasets, such as GTEx, TCGA, and pathway analysis to investigate the role of crocodile serum-induced DEGs in human breast cancer tissue samples. Treated samples revealed 51 differentially expressed spots common to at least two cell lines including proteins involved in cytoskeletal organization and cell death. In this study, for the first time, we report five oncogenes (*TUBA1B*, *SLC2A1*, *PGK1*, *CCND1*, and *NCAPD2*) and two tumor suppressors’ genes (*RPLP2*, *RPL37*) as novel therapeutic targets in breast cancer.

Tubulin Alpha 1b protein, encoded by the *TUBA1B* gene, belongs to the group of tubulin proteins that are a major constituent of the cellular cytoskeleton. These proteins are dynamically involved in the process of cell division and replication [11]. *TUBA1B* was found upregulated in the hepatocellular carcinoma (HCC) and mantle cell lymphoma, also its increased expression was associated with poor survival [12,13]. Very recently, a pseudogene-derived upregulation of the *TUBA1B* gene was also reported in human breast cancer samples [14]. Our TCGA analysis confirmed this as significant upregulation of *TUBA1B* genes in breast cancer tissues, suggesting its potential oncogenic role. Moreover, a genome-wide study reported DNA hypomethylation-induced overexpression of the *TUBA1B* gene in HCC tissue samples [15]. Inconsistent, our analysis showed promoter hypomethylation of the *TUBA1B* gene in breast cancer tissues confirming its epigenetic regulation. Since microtubule proteins play an important role in the regulation of cell division, targeting *TUBA1B* as anticancer therapy could lead to a disruption of microtubules causing cell cycle arrest and apoptosis.

The *SLC2A1* gene provides instructions to encode a protein called the glucose transporter protein type 1 (GLUT1). The GLUT1 protein is a membrane-bound receptor that allows the transport of glucose into cells for use as fuel. The expression of GLUT1 is reported to be significantly elevated in highly aggressive triple-negative breast cancer subtypes [16]. Hence, targeting GLUT1 with novel molecules had been a point of investigation by many researchers [17,18,19,20,21]. Besides, a recent meta-analysis indicated that higher GLUT1 expression is associated with poor prognostic and that GLUT1 might be a potential biomarker and therapeutic target in breast cancer [22]. We showed that the *SLC2A1* gene was hypomethylated and upregulated in human breast cancer tissues. Moreover, the crocodile serum has a strong inhibitory effect on the expression of this gene. Cancer cells require abundant glucose to meet the energy demands of rapidly proliferating cells [22], hence epigenetically silencing *SLC2A1* using crocodile serum provides new strategies for the sensitization of cancer cells to chemo/radiotherapy.

The *PGK1* gene encodes phosphoglycerate kinase enzyme, which is involved in a critical energy-producing process of glycolysis. Recently, PGK1 expression was reported as a part of a 7-gene signature and part of a 4-gene signature to predict the survival of breast cancer patients [23,24]. Furthermore, a higher expression of PGK1 was associated with poor prognosis in breast cancer, as it stimulated breast cancer progression and metastases [25,26,27]. Cancer cells can convert glucose into lactic acid even without a lack of oxygen, glucose is also converted to lactic acid by tumor cells, and the metabolic characteristics of this aerobic glycolysis is called the Warburg effect [22], which has been targeted in the development of imaging and therapeutic drugs.

Non-SMC Condensin I Complex Subunit D2, which is encoded by the *NCAPD2* gene, is a regulatory subunit of the condensin complex. It introduces positive supercoils into relaxed DNA and converts nicked DNA into positive knotted forms. Which is required for the conversion of interphase chromatin into mitotic-like condense chromosomes promoting the process of cell division [28]. We could find only one study that reported that a higher NCAPD2 protein expression was significantly correlated with poor overall survival and worse disease-free survival in triple-negative breast cancer. Also, breast cancer cells transfected with siRNA for *NCAPD2* inhibited its expression and consequently inhibited the proliferation and invasion of cancer cells [29]. We also showed that TCGA breast cancer tissue samples show significantly higher *NCAPD2* expression as well as hypomethylation of its promoter region while we have already reported a crocodile serum-induced significant depletion of *NCAPD2*, which could significantly suppress the proliferation of breast cancer cells probably via failure to complete mitosis.

*CCND1* translates into Cyclin D1, which is an important regulator of the cell cycle. An in silico study has recently suggested that the expression of *CCND1* is positively correlated with hormone receptor positivity in most types of breast cancers [30]. We also found a high expression of *CCND1* in human breast cancer tissues compared to normal. Another study reported miRNA-mediated epigenetic regulation of *CCND1* in breast cancer cells [31]. Here, we showed that DNA hypomethylation might also be mediating the overexpression of this gene and that crocodile serum treatment inducing the silencing of this gene could be beneficial.

*RPLP2* and *RPL37*, both encoding for ribosomal proteins, were identified as tumor suppressor genes due to their silencing in breast cancer tissues. A proteomic study utilizing bioinformatics analysis on breast cancer cell lines identified *RPLP2* among other several genes correlated with chemotherapy-induced cancer cell death [32]. Similarly, in an experimental study on breast cancer patients subjected to neoadjuvant chemotherapy *RPL37* expression along with a few other genes was shown to distinguish responsive from non-responsive tumors [33] suggesting protective roles of both genes. Consistent with this, we found that crocodile-serum induces upregulation of several ribosomal protein-related genes. But the TCGA analysis confirmed hypermethylation silencing of only the *RPLP2* and *RPL37* genes in human breast cancer tissues. Making them ideal positive prognostic markers and crocodile serum as a novel positive regulator of these anti-tumor genes.

In conclusion, we report seven genes that might epigenetically be regulated in breast cancer tissues and could be a potential target for epigenetic therapy. Five of these genes (*TUBA1B*, *SLC2A1*, *PGK1*, *CCND1*, and *NCAPD2*) are thought to be oncogenes, and the two (*RPLP2* and *RPL37*) are believed to be tumor suppressor genes. Also, we believe that the anti-cancer effect of crocodile serum might be due to the induction of promoter methylation changes, specifically in these genes. Epigenetic regulatory pathways comprise an emerging and active area of chemical probe discovery and investigational drug development. Also, there are many great epigenetic drugs, such as 5′Aza-2-deoxycytidine, an inhibitor of DNA methyltransferases in clinical trials for cancer therapy. Prompted by such emerging clinical relevance of epigenetic drugs, we believe that novel molecules present in the crocodile serum could prove to be a potential pleiotropic epigenetic drug, but at the same time have a precise gene target. Further studies on the gut microbiome of the crocodile [1,3] and the sera and gut microbiome of other species that live in polluted environments such as the water monitor lizard, may lead to the identification of novel epigenetic therapeutic targets, given the abilities of these species to be resistant to cancer or infectious diseases, and possess longevity [1,3].

## 5. Conclusions

For the first time in this study, we used a comprehensive in silico approach and identified five proto-oncogenes (*TUBA1B*, *SLC2A1*, *PGK1*, *CCND1*, and *NCAPD2*) and two tumor suppressor genes (*RPLP2*, *RPL37*) as novel therapeutic targets against breast cancer.

## Data Availability

The data that support the findings of this study are available from the corresponding author, NAK, upon reasonable request.

## Supplementary Materials

The following supporting information can be downloaded at: https://www.mdpi.com/article/10.3390/vetsci9050210/s1, Table S1: Details of the 24 genes with CGI in their promoter region and differentially expressed in crocodile serum treated breast cancer cells.

## References

[1] Siddiqui R., Jeyamogan S., Ali S.M., Abbas F., Sagathevan K.A., Khan N.A. (2017). Crocodiles and alligators: Antiamoebic and antitumor compounds of crocodiles. Exp. Parasitol..

[2] Jeyamogan S., Khan N.A., Siddiqui R. (2017). Animals living in polluted environments are a potential source of anti-tumor molecule (s). Cancer Chemother. Pharmacol..

[3] Jeyamogan S., Khan N.A., Sagathevan K., Siddiqui R. (2019). Sera/organ lysates of selected animals living in polluted environments exhibit cytotoxicity against cancer cell lines. Anti-Cancer Agents Med. Chem. Former. Curr. Med. Chem. Anti-Cancer Agents.

[4] Akbar N., Siddiqui R., Sagathevan K., Iqbal M., Khan N.A. (2019). Gut bacteria of water monitor lizard (Varanus salvator) are a potential source of antibacterial compound (s). Antibiotics.

[5] Heerboth S., Lapinska K., Snyder N., Leary M., Rollinson S., Sarkar S. (2014). Use of epigenetic drugs in disease: An overview. Genet. Epigenet..

[6] Dworkin A.M., Huang T.H., Toland A.E. (2009). Epigenetic alterations in the breast: Implications for breast cancer detection, prognosis and treatment. Semin. Cancer Biol..

[7] Muhammad J.S., Guimei M., Jayakumar M.N., Shafarin J., Janeeh A.S., AbuJabal R., Jabal R., Eladl M., Ranade A., Ali A. (2021). Estrogen-induced hypomethylation and overexpression of YAP1 facilitate breast cancer cell growth and survival. Neoplasia.

[8] Jeyamogan S.K.N., Sagathevan K., Siddiqui R. (2020). Crocodylus porosus: A potential source of anticancer molecules. BMJ Open Sci..

[9] Muhammad J.S., Bajbouj K., Shafarin J., Hamad M. (2020). Estrogen-induced epigenetic silencing of FTH1 and TFRC genes reduces liver cancer cell growth and survival. Epigenetics.

[10] Muhammad J.S., Khan M.R., Ghias K. (2018). DNA methylation as an epigenetic regulator of gallbladder cancer: An overview. Int. J. Surg..

[11] Kim N.D., Park E.S., Kim Y.H., Moon S.K., Lee S.S., Ahn S.K., Yu D.Y., No K.T., Kim K.H. (2010). Structure-based virtual screening of novel tubulin inhibitors and their characterization as anti-mitotic agents. Bioorganic Med. Chem..

[12] Lu C., Zhang J., He S., Wan C., Shan A., Wang Y., Yu L., Liu G., Chen K., Shi J. (2013). Increased alpha-tubulin1b expression indicates poor prognosis and resistance to chemotherapy in hepatocellular carcinoma. Dig. Dis. Sci..

[13] Blenk S., Engelmann J.C., Pinkert S., Weniger M., Schultz J., Rosenwald A., Müller-Hermelink H., Müller T., Dandeka T. (2008). Explorative data analysis of MCL reveals gene expression networks implicated in survival and prognosis supported by explorative CGH analysis. BMC Cancer.

[14] Lou W., Ding B., Zhong G., Yao J., Fan W., Fu P. (2020). RP11-480I12.5-004 Promotes Growth and Tumorigenesis of Breast Cancer by Relieving miR-29c-3p-Mediated AKT3 and CDK6 Degradation. Mol. Ther. Nucleic Acids.

[15] Tian Y., Arai E., Makiuchi S., Tsuda N., Kuramoto J., Ohara K., Takahashi Y., Ito N., Ojima H., Hiraoka N. (2020). Aberrant DNA methylation results in altered gene expression in non-alcoholic steatohepatitis-related hepatocellular carcinomas. J. Cancer Res. Clin. Oncol..

[16] Wu Q., Ba-Alawi W., Deblois G., Cruickshank J., Duan S., Lima-Fernandes E., Haight J., Tonekaboni S., Fortier A., Kuasne H. (2020). GLUT1 inhibition blocks growth of RB1-positive triple negative breast cancer. Nat. Commun..

[17] de Castro T.B., Mota A.L., Bordin-Junior N.A., Neto D.S., Zuccari D. (2018). Immunohistochemical Expression of Melatonin Receptor MT1 and Glucose Transporter GLUT1 in Human Breast Cancer. Anticancer Agents Med. Chem..

[18] Hamann I., Krys D., Glubrecht D., Bouvet V., Marshall A., Vos L., Mackey J.R., Wuest M., Wuest F. (2018). Expression and function of hexose transporters GLUT1, GLUT2, and GLUT5 in breast cancer-effects of hypoxia. FASEB J..

[19] Wellberg E.A., Johnson S., Finlay-Schultz J., Lewis A.S., Terrell K.L., Sartorius C.A., Abel E., Muller W., Anderson S. (2016). The glucose transporter GLUT1 is required for ErbB2-induced mammary tumorigenesis. Breast Cancer Res..

[20] Oh S., Kim H., Nam K., Shin I. (2017). Glut1 promotes cell proliferation, migration and invasion by regulating epidermal growth factor receptor and integrin signaling in triple-negative breast cancer cells. BMB Rep..

[21] Zhao F., Ming J., Zhou Y., Fan L. (2016). Inhibition of Glut1 by WZB117 sensitizes radioresistant breast cancer cells to irradiation. Cancer Chemother. Pharmacol..

[22] Deng Y., Zou J., Deng T., Liu J. (2018). Clinicopathological and prognostic significance of GLUT1 in breast cancer: A meta-analysis. Med. Baltim..

[23] Jiang F., Wu C., Wang M., Wei K., Wang J. (2021). Identification of novel cell glycolysis related gene signature predicting survival in patients with breast cancer. Sci. Rep..

[24] Zhang X., Wang J., Zhuang J., Liu C., Gao C., Li H., Ma X., Li J., Sun C. (2021). A Novel Glycolysis-Related Four-mRNA Signature for Predicting the Survival of Patients with Breast Cancer. Front. Genet..

[25] Fu D., He C., Wei J., Zhang Z., Luo Y., Tan H., Ren C. (2018). PGK1 is a Potential Survival Biomarker and Invasion Promoter by Regulating the HIF-1alpha-Mediated Epithelial-Mesenchymal Transition Process in Breast Cancer. Cell Physiol. Biochem..

[26] Sun S., Liang X., Zhang X., Liu T., Shi Q., Song Y., Jiang Y., Wu H., Jiang Y., Lu X. (2015). Phosphoglycerate kinase-1 is a predictor of poor survival and a novel prognostic biomarker of chemoresistance to paclitaxel treatment in breast cancer. Br. J. Cancer.

[27] Shashni B., Sakharkar K.R., Nagasaki Y., Sakharkar M.K. (2013). Glycolytic enzymes PGK1 and PKM2 as novel transcriptional targets of PPARgamma in breast cancer pathophysiology. J. Drug Target.

[28] Ball A.R., Schmiesing J.A., Zhou C., Gregson H.C., Okada Y., Doi T., Yokomri K. (2002). Identification of a chromosome-targeting domain in the human condensin subunit CNAP1/hCAP-D2/Eg7. Mol. Cell Biol..

[29] Zhang Y., Liu F., Zhang C., Ren M., Kuang M., Xiao T., Di X., Feng L., Fu L., Cheng S. (2020). Non-SMC Condensin I Complex Subunit D2 Is a Prognostic Factor in Triple-Negative Breast Cancer for the Ability to Promote Cell Cycle and Enhance Invasion. Am. J. Pathol..

[30] Gan S., Dai H., Li R., Liu W., Ye R., Ha Y., Di X., Hu W., Zhang Z., Sun Y. (2020). Identification of key differentially expressed genes between ER-positive/HER2-negative breast cancer and ER-negative/HER2-negative breast cancer using integrated bioinformatics analysis. Gland. Surg..

[31] Liu Y., Zhang A., Bao P.P., Lin L., Wang Y., Wu H., Shu X., Liu A., Cai Q. (2021). MicroRNA-374b inhibits breast cancer progression through regulating CCND1 and TGFA genes. Carcinogenesis.

[32] Leong S., McKay M.J., Christopherson R.I., Baxter R.C. (2012). Biomarkers of breast cancer apoptosis induced by chemotherapy and TRAIL. J. Proteome Res..

[33] Barros F.M.C., Katayama M.L., Brentani H., Abreu A.P., Barbosa E.M., Oliveira C.T., Góes J., Brentani M., Folgueira M. (2010). Gene trio signatures as molecular markers to predict response to doxorubicin cyclophosphamide neoadjuvant chemotherapy in breast cancer patients. Braz. J. Med. Biol. Res..

[34] Ganesan A., Arimondo B., Rots M.G., Jeronimo C., Berdasco M. (2019). The timeline of epigenetic drug discovery: From reality to dreams. Clin. Epigenetics.

