# Supplementary material for: Crocodylus porosus Sera a Potential Source to Identify Novel Epigenetic Targets: In Silico Analysis
Source: Vet Sci. 2022 Apr 25;9(5):210. doi: 10.3390/vetsci9050210 (PMC9144183; doi:10.3390/vetsci9050210)
Supplement: Supplementary file 1 [file vetsci-09-00210-s001.zip › vetsci-1621982-supplementary.pdf]

Supplementary Table S1: Details of the 24 genes with CGI in their promoter region and differentially expressed in crocodile serum treated breast cancer cells.

Gene	Suggested carcinogenic function	Expression (Crocodile sera treated MCF7)	TCGA expression (Tumor vs healthy)	TCGA methylation (Tumor vs healthy)	Median survival in months *(kmplot.com)*	
					High expression cohort	Low expression cohort
**CCND1**	**Onco**	**Down**	**Up**	Hypo	216.66	228.85
CMC1	TSG	Up	Up	Hyper	39.79	36.9
DDIT4	Onco	Up	Up	Hyper	36	73
EGR1	Onco	Down	Down	No change	216.66	228.85
FTH1	Onco	Up	Up	Hypo	191.21	216.66
HSPA4	Onco	Down	Up	Hyper	41.64	60
HSPA9	Onco	Down	Up	Hyper	43.56	57.3
JUN	Onco	Down	Down	Hyper	216.66	228.85
MTA2	Onco	Up	Up	Hyper	44.5	54.96
MTHFD1	Onco	Down	Down	Hypo	40.56	61
NCAPD2	Onco	Down	Up	Hypo	40.08	60
NCL	Onco	Down	Up	Hyper	44	53.56
PARP1	Onco	Down	Up	Hyper	216.66	228.85
PGK1	Onco	Down	Up	Hypo	30	46
RPL30	Onco	Up	Up	Hypo	48	51
RPL37	TSG	Up	Down	Hyper	44	58
RPLP2	TSG	Up	Down	Hyper	61	41.39
RPS2	Onco	Up	Up	Hypo	46.49	52.54
SCD	TSG	Down	Down	Hyper	58	43.2
SLC2A1	Onco	Down	Up	Hypo	216.66	228.85
TMSB10	Onco	Up	Up	Hyper	191.21	216.66
TUBA1B	Onco	Down	Up	Hypo	41.04	59
TUBB4B/TUBB2C	Onco	Down	Up	Hyper	216.66	228.85
UBC	TSG	Down	Down	Hyper	62	39

(TSG: Tumor-suppressor gene, Onco: Oncogene, Hyper: Hypermethylation, Hypo: Hypomethyation).
